# Registered report: measuring unconscious deception detection by skin temperature

**DOI:** 10.3389/fpsyg.2014.00442

**Published:** 2014-05-23

**Authors:** Anna E. van ’ t Veer, Mariëlle Stel, Ilja van Beest, Marcello Gallucci

**Affiliations:** ^1^Department of Social Psychology, Tilburg Institute for Behavioral Economics Research, Tilburg UniversityTilburg, Netherlands; ^2^University of Milan BicoccaMilan, Italy

**Keywords:** deception detection, physiological markers, indirect deception detection, interpersonal relations, non-conscious perception, skin temperature

## Abstract

Findings from the deception detection literature suggest that although people are not skilled in consciously detecting a liar, they may intuit that something about the person telling a lie is off. In the current proposal, we argue that observing a liar influences the observer’s physiology even though the observer may not be consciously aware of being lied to (i.e., the observers’ direct deception judgment does not accurately differentiate between liars and truth-tellers). To test this hypothesis, participants’ finger temperature will be measured while they watch videos of persons who are either honest or dishonest about their identity. We hypothesize that skin temperature will be lower when observing a liar than when observing a truth-teller. Additionally, we test whether perceiving a liar influences finger skin temperature differently when an individual is, or is not, alerted to the possibility of deceit. We do this by varying participants’ awareness of the fact that they might be lied to. Next to measuring physiological responses to liars and truth-tellers, self-reported direct and indirect veracity judgments (i.e., trustworthiness and liking) of the target persons will be assessed. We hypothesize that indirect veracity judgments will better distinguish between liars and truth-tellers than direct veracity judgments.

## MEASURING UNCONSCIOUS DECEPTION DETECTION BY SKIN TEMPERATURE

Deception is omnipresent and, next to it’s often intended benefits, can have grave interpersonal consequences. A skill for knowing what information to trust is thus an indispensable tool in daily life. Even so, a robust finding in the deception detection literature is that people are no better than chance at detecting a liar (Bond and DePaulo, 2006). If untruths are discovered, they are mostly found out long after the fact or never at all (DePaulo et al., 2004). Yet, research has also shown that people are better able to detect liars when measures are used that do not directly ask them to judge whether someone is lying. Building on evidence suggesting that people are able to differentiate between liars and truth-tellers if asked indirectly, we aim to test the existence of an unconscious indicator (i.e., a physiological marker) of this indirect deception detection. In the current paper, we propose to measure skin temperature, as we believe that this physiological proxy of social interaction could be an important indicator of people’s correct intuition toward liars.

## INTUITIVE DECEPTION DETECTION

As noted above, people are not very good at verbalizing whether another individual is lying or not. Indeed, people’s direct veracity judgments rarely exceed what could be expected on the basis of chance (Bond and DePaulo, 2006). Yet when people’s judgment of a liar is assessed in an indirect way, they do seem to be able to distinguish the liar from the truth-teller. Although this does not mean that people are aware that they are being lied to, it does mean that compared to truth-tellers, people’s impressions of, or feelings toward, liars are different. One telling piece of evidence for people’s ability to indirectly detect deception is a study comparing direct (“Is the person lying?”) to indirect (“Does the person have to think hard”) judgments made by police officers (Vrij et al., 2001). Results of this study indicate that the *indirect* judgments distinguished between liars and truth-tellers (i.e., the liars were judged to be thinking harder), whereas the *direct* judgments did not. 

A meta-analysis touching upon indirect deception detection also found that people report more confidence in their judgment after perceiving a truthful compared to a dishonest message (DePaulo et al., 1997), leading authors to conclude that this supported the idea that feelings of confidence—as indirect measures of deception detection—might differentiate truths from lies. Additionally, subjective impressions seem to distinguish liars from truth-tellers better than objective measures. In their meta-analysis, DePaulo et al. (2003) found that subjective measures of verbal immediacy (e.g., active vs. passive voice), eye contact, and facial pleasantness all discriminated between a liar and a truth-teller, whereas the objective measurements of these features (e.g., the coding of their occurrences by independent researchers) did not. The most compelling evidence for people’s ability to sense someone is lying comes from research comparing an intuitive to a more deliberative processing style. Albrechtsen et al. (2009) found that intuitive judgments of deception were more accurate than deliberative judgments. On top of this, these authors found that automatic judgments made when conscious attention was directed at a concurrent task were more accurate than judgments made after conscious reasoning about ones deception judgment. These findings suggest that, on some level, people intuit that they are being lied to while they are forming an impression of a liar.

The fact that directly judging someone to be a liar is difficult is understandable considering that there is a lack of cues that people can use to reliably detect a liar (DePaulo et al., 2003). Yet even though cues might be weak, some evidence of their presence does exist. Liars are perceived as more tense and less forthcoming, they have less compelling stories, speak in a higher pitch, and make a more negative impression—truth-tellers, on the other hand, come across more direct, certain, and more personal (DePaulo et al., 2003). Although not always consciously aware of it, people are very good at picking up subtle cues from their social environment (Bargh and Chartrand, 1999). This is, for instance, supported by both research on mimicking suggesting that non-verbal behavior is regulated mostly outside of conscious awareness and that it has consequences at the behavioral level (Chartrand and Bargh, 1999; Stel et al., 2008), and research on emotional face-to-face communication (Dimberg et al., 2000). Similarly, as we suggest here, people might thus unconsciously pick up on some of the less apparent cues given away by a liar.

Our reasoning is based on indications that forming impressions of the intentions of other people seems to be an automatic process, one that has been argued to be evolved in order to enhance chances of survival (Fiske et al., 2007). More specifically, to the extent that forming alliances with trustworthy others benefits survival and reproduction, being able to detect trustworthiness in others has adaptive value. One major marker for trustworthiness is emotional expressivity, where emotional expressiveness is positively related to being judged as trustworthy (Boone and Buck, 2003). As liars may try to control their expressive behaviors (DePaulo et al., 2003), liars could generally be perceived as less trustworthy. People judge trustworthiness of others very rapidly (Willis and Todorov, 2006), and base their social decision-making on it (van ’t Wout and Sanfey, 2008). People are also especially good at judging someone’s warmth—an indication of the favorability of another persons’ intentions toward us—as compared to their competence (Fiske et al., 2007). In similar vein, people judge liars less likable and less trustworthy than truth-tellers, and tend to increase their own deceptive behavior toward a liar (Tyler et al., 2006). It appears that people are wired to detect friendly intent or potential threats in others, and adjust their behavior toward them accordingly.

Seminal work has demonstrated that from early on in life, being able to know who to trust and forming emotional attachments is essential for development (Bowlby, 1969), and that physical contact is essential for survival and psychological wellbeing (Harlow, 1958). Accordingly, it has been argued that the association between warmth and trust is strengthened during early development, as physical warmth usually co-occurs with care from trusted others (IJzerman and Koole, 2011). Recent research also suggests there is a relationship between perceiving a person as trustworthy and temperature perceptions. Szymkow et al. (2013) found focusing on traits unrelated to trustworthiness did not effect perceptions of ambient temperature, whereas focusing on traits relevant to trustworthiness (i.e., communion, warmth) did. This also led the authors to argue that perceptions of temperature—which could arguably be stemming from bodily temperature changes—can inform on the trustworthiness of others. The question we are concerned with here is whether forming an impression of an untrustworthy or trustworthy person influences actual physical temperature.

## PHYSICAL AND INTERPERSONAL WARMTH

There have been a number of studies linking skin temperature to interpersonal relations. For instance, social exclusion not only makes people feel bad but it also makes them feel colder (Zhong and Leonardelli, 2008) and this is reflected in actual skin temperature (IJzerman et al., 2012). Correspondingly, temporarily holding a warm object—such as a tea cup—can mend this negative affect (IJzerman et al., 2012) and positively influences judgments of interpersonal warmth (trust) and enhances positive behavior toward others (Williams and Bargh, 2008). Physical temperature has also been found to influence trust behavior in an economic trust game (Kang et al., 2011), and as mentioned above, focusing on a target persons’ psychological warmth increases estimates of ambient room temperature (Szymkow et al., 2013). These findings suggest a process of bodily temperature regulation during social interaction, wherein elements of the interaction influence the body and *vice versa*. Building on these ideas of the embodiment of social relationships, the question arises, then, whether unconsciously picking up on untrustworthiness (i.e., another person lying) is also accompanied by temperature changes.

Finger skin temperature is an excellent way of assessing psychophysiological change and reflects sympathetic vasoconstriction—a known reaction to pain or mental and emotional stressors—with an average delay of about 17 s (Kistler et al., 1998). Although in the case of thermoregulation a lot is still to be revealed about its causes and consequences, there are some notable findings. For instance, a decrease in skin temperature is usually associated with negative or stressful events, such as being asked threatening personal questions (Rimm-Kaufman and Kagan, 1996), anticipating and receiving electric shocks (Boudewyns, 1976), watching the shower murder scene of Alfred Hitchcock’s movie “Psycho,” hearing the noise of a ruler slapping on a table without seeing it (Kistler et al., 1998) or being excluded (IJzerman et al., 2012). It is also found that the decrease in finger skin temperature that is observed during relatively stressful events can be alleviated by a subsequent relaxation phase (Boudewyns, 1976). Obviously, stressful experiences are not the only elicitor of changes in physiology, and with this work we aim to expand knowledge on the aforementioned relationship between the social environment and thermoregulation.

We suggest that to capture the full range of peoples’ reactions to liars, the physiological reaction of the observer of a liar should also be taken into account. Whereas we propose to focus on the observer of a liar, to date, more is known about the physiology of liars themselves (cf. Podlesny and Raskin, 1977; Wang et al., 2010). For instance, in interrogation settings, the polygraph is a well-studied instrument, but it is also far from perfect (see Lykken, 1998, for a critical review). More specific to thermoregulation, the stress in a sender of a deceptive message is found to manifests itself in blood flow to the face (specifically, the orbital muscle area) resulting in elevated temperature in this area (Tsiamyrtzis et al., 2006). However, to our knowledge, we are the first to put forth the argument that the physiology—and in particular the thermoregulation—of the receiver of a deceptive message should be investigated to acquire knowledge of the underlying mechanisms of social interactions.

## THE PROPOSED RESEARCH

In the proposed study, we aim to explore whether skin temperature is influenced by observing liars and truth-tellers and whether temperature relates to self-reported judgments of these liars and truth-tellers. Previous work has provided two notions that are of interest to the current thesis: physiological markers can precede explicit knowledge (Bechara et al., 1997), and, these markers influence decision-making (Bechara and Damasio, 2005, but see Dunn et al., 2006, for a critical evaluation). In the case of deception detection, a physiological marker may precede explicit judgment of a liar. To our knowledge, no attempt to find such a physiological marker of deception detection exists to date. Yet if this process by which physiological markers influence peoples’ deception judgment could somehow aid people’s conscious assessment of a liar, it does not seem to do so unless they are induced to rely on their intuition (Albrechtsen et al., 2009). For this reason, we will ask participants to rate both their liking and trustworthiness of liars and truth-tellers, as *indirect* measures, and their *direct* veracity judgment. We will measure this together with finger skin temperature in two distinct situations: first when participants are *not* aware they might be lied to, and subsequently, when they *are* aware of this possibility. We thereby examine whether forming an impression of a liar compared to a truth-teller, even when not having a conscious goal of detecting deception, is accompanied by physiological states that can be differentiated by measuring finger temperature.

Below we first describe an exploratory pilot study that was conducted to familiarize ourselves with methods of investigating the proposed association between finger skin temperature and unconscious deception detection. We then describe how and why this pilot study can be improved, and then we elaborate on an experiment that we propose to conduct which incorporates these improvements.

## PILOT STUDY^1^

In the pilot study there were 132 participants. Applying the same exclusion criteria for the proposed experiment that is described below, 18.2% of participants was excluded (1 for failure to save the data, 21 for recognizing a target person, 2 for participating before) leaving 108 participants (*M*_age_ = 20.62, *SD*_age_ = 2.49, 64.8% female).

In the pilot study, participants first saw a 5-min long neutral nature movie and then saw a total of four videos of 3 min each in which a target person either gave a truthful or an untruthful impression of themselves (below we refer to this as the target person’s veracity). While participants were watching these videos, we measured their finger temperature. The four videos in which a target person either lied or told the truth were structured in two blocks of two videos each; in the first block participants randomly saw one truth-teller and one liar, and in the second block they randomly say one truth-teller and one liar. After the first block of videos participants answered two dependent measures for both videos in the first block, and they again answered the same measures after the second block. This allowed us to ensure that for the first two videos the participants were not aware of the fact they might be lied to, but for the last two videos they were (below we refer to this factor as awareness). For each target person, the participants were asked to indicate how much they liked this person, and whether they thought the target person was telling the truth (both on 7-point scales)^2^.

### RESULTS OF THE PILOT STUDY

Concerning the temperature data, first a linear mixed model was performed on the pilot data, considering the experimental factors and linear and quadratic time as both fixed and random effects. In order to estimate the average linear trend of time and to ease the parameter interpretation, time has been centered to 85 s, as the overall time of each video was 170 s. The intercept (σ = 0.04, Wald *Z* = 7.24, *p* < 0.001), the effect of awareness (σ = 0.19, Wald *Z* = 7.34, *p* < 0.001), the effect of veracity (σ = 0.13, Wald *Z* = 7.33, *p* < 0.001), the linear effect of time (σ < 0.001, Wald *Z* = 7.28, *p* < 0.001), the interaction between veracity and awareness (σ = 0.57, Wald *Z* = 7.33, *p* < 0.001), and the quadratic term of time (σ < 0.001, Wald *Z* = 7.09, *p* < 0.001) varied randomly across participants. These results showed that participants had different intercepts, that they differed in the intensity with which awareness and veracity affected their temperature, that they were differently affected by veracity in different awareness phases and that their linear and quadratic trends over time differed.

In our full model, regarding the fixed effects, there was a three-way interaction between veracity, awareness and time, *b* = 0.0002, *F*(1,73218.7) = 6.69, *p* = 0.010. We also found an average (across time) interaction between veracity and time, *b* = 0.0007, *F*(1,73218.7) = 387.14, *p* < 0.001, an average (across time) interaction between awareness and time *b* = -0.001, *F*(1,73218.7) = 993.88, *p* < 0.001, and a quadratic effect of time, *b* = 0.000008, *F*(1,107.430) = 14.04. Additionally, there was a significant main effect of awareness, *b* = -0.107, *F*(1,108.105) = 6.78, *p* = 0.011. There were no significant main effects of veracity, order, and time. Order did not interact with the other experimental factors, and there was no interaction between veracity and awareness, all *p*’s > 0.175.

**FIGURE 1:**
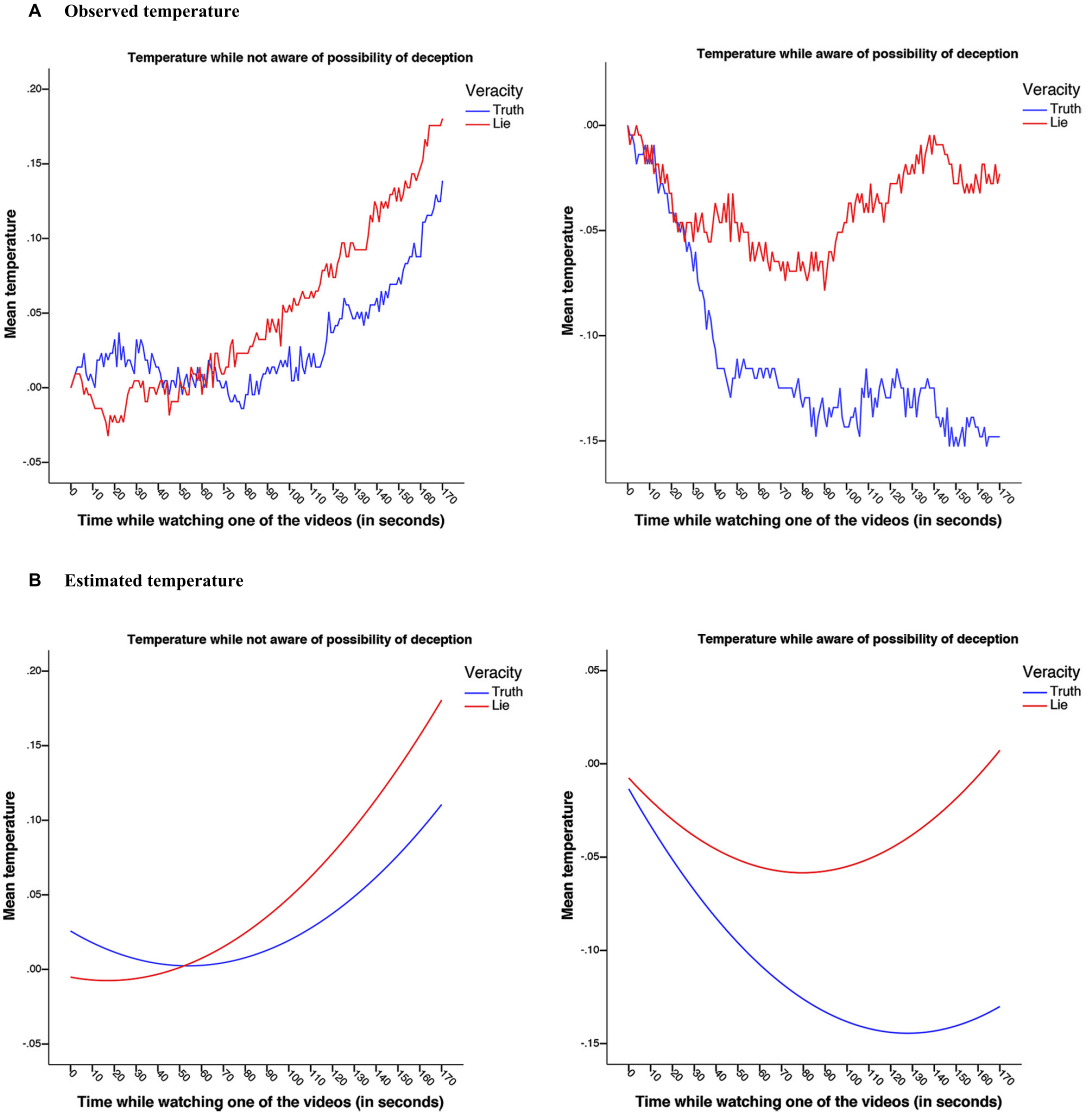
**Average observed (A)** and estimated **(B)** temperature change over time in the pilot study as a function of type of video, broken down by awareness levels.

These results suggest that veracity predicted temperature differently in the unaware vs. the aware phase, and that this effect was changing over time. To understand the interactions, we plotted the average temperature over time as a function of veracity, broken down by awareness levels (see **Figure 1**).

From **Figure 1**, it becomes apparent that in the not aware condition (i.e., in the first block of videos) participants were warming up over time while watching both a video of a liar or a truth-teller. Interestingly and surprisingly, in the second block of videos (what we have called the aware phase) finger temperature dropped when watching a video of a truth-teller, more so than when watching a liar.

In the pilot study, participants’ liking of the persons on the videos was assessed (*indirect* judgment), and whether they felt that the person was telling the truth (*direct* judgment). Liking for liars was lower (*M* = 4.57, *SD* = 1.00) than liking for truth-tellers (*M* = 5.15, *SD* = 0.80), *t*(107) = -4.873, *p* < 0.001, 95% CI [-0.81, -0.34], Hedges’s *g*_av_ = 0.64. The CL effect size indicates that after controlling for individual differences, the likelihood that a participant scores a truth-teller higher on liking than a liar is 68%. The direct deception judgment, however, was not significantly different for liars (*M* = 5.10, *SD* = 1.14) than for truth-tellers (*M* = 5.10, *SD* = 1.14), *t*(107) < 1, *p* = 1. This indicates that even though participants disliked liars more than truth-tellers, they did not explicitly judge them as being more deceptive than the truth-tellers. We conclude from this that people are indeed able to intuit that something about a liar is “off” and sense a subtle difference between liars and truth-tellers when their judgment is assessed in an indirect way.

Correlations were run between temperature and liking and temperature and veracity judgment for liars and truth-tellers. Out of eight correlations run (temperature during a first block-truth with both the direct and indirect judgment of the truthful person, temperature during a first block-lie with both the direct and indirect judgment of the liar, and the same four correlations for the second block) there were two significant correlations, both in the second block: the higher a participant’s temperature while watching a truth-teller, the more this participant thought the target person was telling the truth, *r*(106) = 0.26, *p* < 0.01, and the more this participant thought the target person was likable, *r*(106) = 0.19, *p* < 0.05.

To summarize, in the pilot study we found that participants’ finger temperature increased while watching a liar or a truth-teller in the first block of videos (i.e., when participants were not aware of the possibility of being lied to), but in the second block of videos (i.e., when participants were aware of the possibility of being lied to), we found participants’ finger temperature dropped while watching a liar or a truth-teller, but more so for the truth-tellers. This interaction of veracity with the different experimental phases is interesting in itself, as it shows that our design and stimuli do indeed elicit temperature changes in our participants.

Findings of the pilot study were, however, not consistent with the reasoning outlined in the introduction, and therefore we feel the proposed study will provide a unique opportunity to confirm or disconfirm our hypothesis that participants’ finger temperature will lower when perceiving a liar. In the pilot study, participants’ temperature was still increasing with time during the first videos a participant encountered (irrespective of whether they were watching a truth or a lie). The neutral movie in the pilot study might thus have been too short for participants to reach a stable temperature. We feel we can improve on the study’s procedure by allowing a longer “warm up time.” In other words, for the proposed study, we will present the participant with a longer neutral movie before the target persons are presented. Results of the pilot study also indicate that participants could differentiate between liars and truth-tellers on the indirect veracity judgment (i.e., liking), but not on the direct veracity judgment. In the proposed study, we aim to replicate this finding to assess the robustness of this effect of indirect deception detection, and we add to it by now also incorporating a measure of trustworthiness of the target person. These direct and indirect veracity judgments also allow us to replicate the finding in our pilot study that higher finger skin temperatures correlate with the judgment that someone is telling the truth and the liking of that person. In our opinion, these initial results provide a tentative indication of a physiological marker intertwined with a mechanism designed for (unconscious) deception detection.

## PROPOSED EXPERIMENT: AIMS AND HYPOTHESES

The aim of this study is to assess whether finger skin temperature differs when watching a liar as apposed to a truth-teller. We thereby combine the deception detection literature with the growing literature on embodiment. Our main hypothesis is that temperature will lower during the watching of a 3-min video clip of a liar (H1). We further hypothesize that participants will judge truth-tellers more trustworthy and likeable than liars (the *indirect* veracity judgments; H2a), with the additional hypothesis that this effect will be bigger for the trustworthiness judgment than for the liking judgment, because trustworthiness judgments are suggested to be more automatic and intuitive and would therefore tap into the covert differences between liars and truth-tellers better (H2b). Next to this, we hypothesize that when asked to judge whether a target person is lying, participants’ judgment will not differentiate between liars and truth-tellers better than chance (the *direct* veracity judgment; H3). Finally, we hypothesize that the indirect veracity judgments, namely the liking and trustworthiness for the target person, are positively related to finger temperature, whereas the direct veracity judgment is not (H4). Additionally, our proposed design allows us to infer whether these effects interact with the level of awareness participants have of the fact that this is a setting in which deception has to be detected.

We regard this registered report as a unique opportunity to shed light on findings that were obtained in our pilot study. We aim to improve on the methods used in the pilot study by now also including a measure of trustworthiness, and by measuring the direct veracity judgment with a binary choice option, in order to be able to compare our participants’ performance in detecting deception to performance on the basis of chance (see H3). We aim to replicate the findings of the pilot study that truth-tellers get more positive judgments on *liking* (and for the proposed study, also on trustworthiness, see H2a) and that finger skin temperature is positively correlated with this (see H4). Most importantly, we aim to better test our prediction that we make on the basis of thermoregulation in (dis)trusting interactions that we will observe a lowering in finger temperature when watching a person who is dishonest compared to honest (see H1), by now allowing more time for participants to reach stable temperature.

## METHOD

### DESCRIPTION OF PROPOSED SAMPLE CHARACTERISTICS

The proposed sample will consist of the Tilburg University lab participants, a sample that on average consists of about 65% females, 95% University students who are mainly Psychology undergraduates around the age of 21, who participate for course credit or an hourly pay of €8. We propose to run at least 120 participants (see below). We will apply several exclusion criteria. First, participants who have prior experience with the temperature measure (and its debriefing) will be offered participation in another study and will be refused participation in the current study on theoretical grounds, as people may be able to consciously control their own finger temperature (Keefe, 1978). Second, participants will be excluded from analyses if they are acquainted with one or more of the people depicted in the video material (assessed after all dependent measures). This is done because knowing the target person in this case will almost always result in being able to tell whether what the person is saying is true (e.g., recognizing someone from a psychology class while this person faints an education in another area). Third, participants will be excluded in the following instances: technical failure of temperature measurement, defined as either a software error, crashing of the computer program or a failure on the experimenter’s part to correctly start measurement or save it (in which case no temperature recording is present for this participant, yet other dependent measures may be present).

Contrary to what was done in previous research (IJzerman et al., 2012), we will also run exploratory analyses without participants who fail to reach 30°C during watching of the neutral movie, as some authors have argued that fingertip temperature should be high enough to observe vasoconstriction (i.e., it should be physiologically possible; Kistler et al., 1998). The analyses including these participants are the main analyses. Analyses will also be run both with and without heavy smokers (more than 20 cigarettes a day) as smokers can have trouble warming up after cooling down (Cleophas et al., 1982), the analyses without them being conclusive. Outliers are defined as having an unlikely to be correct bodily temperature, cut offs set at below 18°C and above 37°C. If outliers are present in the data, we will employ a jackknife methodology to confirm the robustness of the results and report differences in outcomes in a footnote.

### PROCEDURE

Possible participants will enter a draftless lab room with a maximum of 12 at a time. They will be led to an individual table with a computer separated by screens, where they sign for informed consent. A maximum of six participants will be participating in the current study at a given time. After completing half an hour of unrelated tasks (to allow for acclimatization to the room temperature), the experimenter will set up the current experiment run in both Authorware and, for the temperature measure, OneWireViewer. Participants’ finger skin temperature will be measured with a so-called iButton (see Pouw et al., 2012, for software and instructions), introduced to the participant as “a battery measuring a physiological response.” The iButton will record temperature every second. The iButton clock will be synchronized to system seconds, and the start and end time of all videos shown will be saved in Authorware (which also relies on system seconds to retrieve the current time). During the time the experimenter starts the temperature log, the participant will be asked to clean their fingers with an antibacterial wipe and to indicate which is their dominant hand. After this the experimenter will attach the iButton to the palmar surface of the distal phalange of the non-dominant index finger with a double-sided EEG sticker. The participant will then be instructed to comfortably lay their forearm on the table with the iButton facing up, and to start working through the experiment in Authorware.

All participants will first see part of a nature documentary for a minimum of 8 min allowing for the iButton to reach finger temperature, and for the participant to reach a stable starting temperature. Two filler questions about this documentary will be asked to seemingly give the documentary a purpose and to acquaint the participant with the overall procedure of watching a video and subsequently answering questions about it. Next, the participants will be told they will now watch a series of different videos. They are explicitly informed that these videos will be presented in blocks and that questions will follow after each block. Each block contains two videos and thus after two videos questions will be asked about the person in both videos. The participant is not told the total number of videos that will follow, to minimize their possible tendency to expect 50% of the videos to be untruths. In reality, four videos will be shown in total (see Video material), divided in two blocks of two videos. Each block consists of one liar and one truth-teller, randomly presented. The gender of the person on the video will also be varied to make sure lies are not confounded with gender.

After each block of two videos, the participant will answer three questions about each video. Firstly the *indirect* deception judgments are assessed with two counterbalanced questions: “How much do you like the person in the first [second] video?” and “How trustworthy did you think the person in the first [second] video was?”, responses will be given on a 7-point scale, ranging from 1 (*not at all*) to 7 (*very much*). Thirdly, the *direct* deception judgment is assessed with the question: “Did you think the person in the first [second] video was telling the truth?” (*yes* vs. *no*). Note that the nature of the questions about the target persons is thus revealed after block 1, and that the participant now knows that the possibility exists that the person in the video is insincere. To strengthen this manipulation of the level of this awareness, participants are told that for the next block of videos, they will receive the same three questions about the target persons. The three questions will also be presented to the participants again in order to make sure they realize what they will be asked after the next videos. This allows us to compare our dependent measures in both blocks to examine the effect of this awareness of possible deception. Lastly, the participants will provide information on their gender, age, smoking behavior, acquaintance with any of the people depicted in the videos, dominant hand, and their thoughts on what the study was about. The iButton will then be detached and the participant will be thanked and debriefed.

### VIDEO MATERIAL

Videos used in this study will display two men and two women who have each been recorded separately while they introduce themselves for 3 min, either truthfully or deceptively, making eight videos in total^3^. These persons were instructed to give an impression of themselves, talking about topics like their personality, interests, family situation, childhood, education, and work situation. As people frequently lie to make a good impression, for instance in job interviews (Weiss and Feldman, 2006), impression management is a topic particularly relevant to the current study. Additionally, it has been found that both men and women who have a self-presentation goal (i.e., to appear likable or competent), compared to those who don’t have this goal, lie significantly more in real-life interactions (Feldman et al., 2002). In our opinion, this makes having to form an impression of a person on a video—who is trying to give a good impression of themselves—an appropriate setting in which to examine the participants’ physiological responses to deceit. Target persons on the videos read instructions that told them they were randomly assigned to tell the truth first, and then lie, to another participant (who, in fact, was a confederate). They were instructed to talk about themselves for 3 min truthfully, and were told that 3 min would feel like a long time, so they should try to give a complete picture of different aspects of themselves. They directed their speech to the confederate, who sat next to the camera. After giving this true impression the confederate briefly left the room, supposedly to fill out a questionnaire. During this time, the target person was instructed to give an impression of themselves for a second time when the confederate returned, while this time being untruthful. The confederate then came in again for this second recording, and was supposedly going to guess which one of the two times an impression was given would be the truth.

### PROPOSED ANALYSIS PIPELINE

The following pre-processing steps will be taken. First all individual temperature data files will be jointly imported to SPSS with “TempToSPSS,” a piece of software custom programmed by SpITs, the Tilburg University IT department. Authorware data will also be joined and subsequently these two datasets will be combined. As the Authorware dataset will contain a variable indicating the time of a given video and the order in which the program randomly displayed the videos, new variables can be made indicating at what point in time (thus belonging to which temperature data) which of the nine videos was displayed to the participant (neutral nature video clip or one of the eight videos of four persons lying or telling the truth), and whether a liar or a truth-teller was in it. Next, a new time variable will be made that starts at 0 for each new video encountered by the participant, allowing the temperature data to be displayed and analyzed over time, collapsing over videos (all SPSS syntax steps will be made openly available online). For each video that a participant is watching, we will compute the participants’ temperature minus their temperature at the beginning of this video, in order to ensure that any differences between participants’ temperature at the beginning of the video will not influence the outcomes. This way, we make sure that individual carry-over effects from the last video that was watched are kept to a minimum.

In the statistical models described below we refer to the experimental factors in the following way: the factor veracity, representing whether the person on the video was lying or not, the factor awareness, representing whether the participant was aware of the possibility of being lied to or not, and the factor order, representing whether a liar was shown first and then a truth-teller, or *visa versa*.

To analyze the indirect veracity judgments, to test whether truth-tellers compared to liars get higher judgments (see H2a) we will estimate separate linear mixed models, one with liking and one with trustworthiness as the dependent variable, and with the experimental factors veracity, awareness and order as independent variables. To analyze the direct veracity judgment (“Is this person lying, yes or no?”) to test whether truth-tellers compared to liars get different judgments we will estimate a mixed logistic model with this binary variable as the dependent variable and veracity, awareness and order as independent variables. The same model will allow us to test whether the judgments in each experimental phase deviate from chance (i.e., equal probability), thus providing a test of accuracy (see H3). Furthermore, we will compute a variable indicating whether the participant made a correct direct veracity judgment for each video. A mixed logistic model will be used to assess the relationship between this indication of accuracy and the participants’ mean temperature per video across different experimental phases (unaware vs. aware). Using a random coefficients regression we will also assess the relationship between temperature and both liking and trustworthiness (see H4).

The main statistical analyses’ aim is to estimate the effects of veracity of the person on the video (lie vs. truth; see H1) and deception-possibility-awareness of the participants (aware vs. not aware) on participants’ temperature. In order to ease interpretation, we will center time as was done for the pilot study. Using linear mixed models (SPSS mixed), a model will be built to define the trajectory of temperature over time (with linear and polynomial effects of time), as a function of veracity and awareness. Order of both the independent variable (veracity) and the dependent variables (liking and trustworthiness) will be considered in the analysis with its main effect and interactions with the experimental factors. All repeated measures effects—intercept, polynomial time, awareness, and veracity will be allowed to vary randomly, removing those effects that show no variability across participants. Variances and covariances will be tested with Wald tests against the null hypothesis of no variance, but any parameter greater than 0 will be left as random (Littell et al., 2000). When the random components are ascertained, we will estimate the complete model using restricted maximum likelihood. Satterthwaite approximation of the degrees of freedom will be used (West, 2009). Specifically, this full model has ID (participants) as subjects variable, includes temperature as the dependent variable, and time as the continuous (polynomial) independent variable, veracity, awareness and order as predictors to the model. This model estimates the main effect of type of video, the main effect of deception-possibility-awareness, the main effect of order, their interactions and the interaction of the experimental factors with time. This last effect informs on whether the temperature trajectories change depending on the veracity and awareness. Order of the videos is added to the model to account for any order and carry over effects; if order interacts with the experimental factors we will discuss implications of this for the validity of the results, if order has a main effect, taking this variable into account strengthens the models’ statistical power. The most important effects these models allow us to estimate are the interaction effects of our experimental factors and time, because the expected change in temperature due to the experimental factors should unfold over time. For exploratory purposes, we will run our full model with gender as a fixed predictor to see whether it interacts with any of the experimental factors (if this reveals an interaction with the experimental factors, we will leave gender in the model that we report in our results section).

In short, we will regard H1 as confirmed if the average skin temperature of participants while watching a liar is lower than when watching a truth-teller. This should translate into an interaction between veracity and time, and possibly a main effect of veracity. We will regard H2a as confirmed if liking and trustworthiness are significantly higher for the truth-tellers compared to the liars, and H2b as confirmed if the effect size of this effect is larger for trustworthiness than for liking. We will regard H3 as confirmed if the overall proportion of correct veracity judgments made by the participants is not significantly different from equal probability or if it is significantly different from equal but lower (due to truth-bias). We will regard H4 as confirmed if liking and trustworthiness are significantly positively related to temperature and partly confirmed if either one of these indirect measures is, whereas accuracy (on the direct veracity judgment) is not.

### STATISTICAL POWER ANALYSIS

For temperature measurements in experiments about social relations, effect sizes in the literature are scarce, although some do exist. For instance, IJzerman et al. (2012) found a *B* of -0.011 for the effect of being excluded during an experiment that employed a ball-tossing game. As our proposed stimuli are hypothesized to elicit an unconscious reaction, we argue the change in fingertip temperature could be even smaller than this, and therefore we aim to have as many participants as possible. For our design and our specific needs we were not confident in proposing an appropriate way of determining sample size. For this reason we propose to go beyond the sample size suggested for a between-subjects design, namely the suggestion for the rule of thumb to have a minimum of 50 participants in each condition (Simmons et al., 2013). With our exclusion criteria in mind, we set out to run a minimum of 120 participants. Note that the above-mentioned rule of thumb is based on a between-subjects design, and our design repeatedly measures participants’ temperature for both lies and truths, thus is a within-subjects design. This means we will collect a minimum of 160 temperature observations (measured each second of video material) four times per participant (during the first and second lie and first and second truth). Thereby, this design increases the probability of finding a finger temperature difference between perceiving truths and perceiving lies if one exists.

### TIMELINE FOR COMPLETION OF THE STUDY

After In Principle Acceptance (IPA), this study can commence within 1–3 weeks provided that this is not during major holidays. We estimate the study will run for 2–4 weeks in order to achieve the desired sample size.

## Conflict of Interest Statement

The authors declare that the research was conducted in the absence of any commercial or financial relationships that could be construed as a potential conflict of interest. The Editor declare that while the authors Anna E. van ’t Veer, Mariëlle Stel, Ilja van Beest are currently employed by the same institution (Tilburg University, Department of Social Psychology) there has been no conflict of interest during the review and handling of this manuscript.

## Footnotes

1 Data and accompanying syntax can be found via the link: http://openscienceframework.org/project/bgrVB/node/YZjUB/files

2 Because the procedure of the pilot study and the proposed study are identical (with the exceptions that we did not measure trustworthiness in the pilot study, that the “warm up” time was shorter in the pilot study, and that we manipulate awareness less strongly in the pilot study), we refer to the description of the proposed study for a detailed account of the procedure and materials used.

3 Notice that of the eight videos, a given participant is only shown four. This ensures that participants do not see a lie and a truth of the same target person.
